# Musical Hallucinations and Forgotten Tunes – Case Report and Brief Literature Review

**DOI:** 10.3389/fneur.2013.00109

**Published:** 2013-08-08

**Authors:** Danilo Vitorovic, José Biller

**Affiliations:** ^1^Department of Neurology, Stritch School of Medicine, Loyola University Chicago, Maywood, IL, USA

**Keywords:** musical hallucinations, auditory processing, memory

## Abstract

Musical hallucinations represent a specific form of auditory hallucinations whereby patients experience formed music without an external source. We describe a 60-year-old woman with history of bilateral hearing impairment and tinnitus who experienced both recognizable and non-recognizable songs. Curiously, she was able to reproduce non-recognizable songs in a way that could be recognized by others. This phenomenon is in line with current understanding that musical hallucinations represent abnormal activity in the auditory associative cortices, raising intriguing questions regarding memory, forgetting, and access to lost memories.

## Introduction

Musical hallucinations represent a specific form of auditory hallucinations whereby patients experience formed songs, instrumental music, or tunes, without an external musical stimulus. The prevalence of musical hallucinations in the general population is largely unknown due to its rarity, and also likely due to patients’ underreporting. The prevalence of musical hallucinations at a general hospital setting has been estimated to be 0.16 (1), and 2.5% among elderly subjects with audiological complaints (2). Hermesh showed that 26.8% of outpatient psychiatric patients reported musical hallucinations, while the prevalence rose to 41% among patients with obsessive-compulsive disorder (3).

Musical hallucinations often present as well-known tunes in the context of patients’ culture, and more often occur among elderly women with underlying hearing impairment (4). Most patients have insight into their condition and perceive the musical hallucinations as intrusive and occasionally unpleasant.

We evaluated a middle-age woman with history of bilateral hearing impairment and tinnitus that experienced both recognizable and non-recognizable songs, and was able to reproduce non-recognizable songs in a way that were recognized by family members as popular songs. To our knowledge, this is the first report of musical hallucinations of non-recognizable songs that were recognized by others in the patient’s environment. This raises intriguing questions about musical memory, as well as mechanisms of forgetting.

## Case Report

A 60-year-old woman with history of bilateral sensory-neural hearing loss for a few years, presented to our clinic for a second opinion regarding her musical hallucinations. Her musical hallucinations developed suddenly, one evening while trying to go to sleep, and approximately 10 months prior to presentation in our clinic. Her hallucinations were described as music playing from a radio at the back of her head, without further lateralization. She heard parts of popular songs, consisting of musical excerpts with well-formed vocals and accompanying instrumental music. There were songs she was able to recognize, but there were also songs she could not. However, she was able to sing parts of the songs or hum the tunes she experienced during her hallucinations, while her husband was often able to recognize those as popular songs. All of the songs she heard at some point in her life. Initially, she perceived the music only at night time while attempting to fall asleep, but within 4 months after onset, the musical hallucinations were present all the time. At times, one song would play repeatedly for 3 weeks before being substituted by another song. There was no variation in loudness. The patient was able to hear and follow conversations in her environment. Concurrent exposure to different tunes would not influence her musical hallucinations. She did not experience non-musical auditory hallucinations, visual, tactile, or olfactory hallucinations. She also had some dull frontal headaches and insomnia that at times interfered with daily activities. Eight years prior to presentation, she recalled onset of a rather monotonous bilateral tinnitus, and 3 years prior to presentation, she was diagnosed with mild to moderate sensory-neural hearing loss. She was advised to have hearing aids which she did not obtain due to apparent financial constraints. There was no history of head trauma changes in mood, energy, appetite, or cognitive functioning. Prior trials of clonazepam, quetiapine, and zonisamide did not improve her symptoms, and were subsequently stopped due to side effects. Neurologic examination was unremarkable except for bilateral, symmetric sensory-neural hearing impairment.

Complete blood count, complete metabolic panel, gamma-glutamyl transpeptidase, C-reactive protein, erythrocyte sedimentation rate, rheumatoid arthritis screen, serum vitamin B12, serum folate, iron, plasma homocysteine, and vitamin D levels were normal. Anti-nuclear antibody was positive at 1:80 dilution. Magnetic resonance imaging (MRI) of the brain with and without contrast was normal. Positron emission tomography (PET) scan was unremarkable. A 24-h video electroencephalogram (EEG) was unremarkable. Audiogram demonstrated moderate sensory-neural hearing loss. Video nystagmography showed paroxysmal up and left beating nystagmus both with right and left ear undermost with Dix–Hallpike testing, suggestive of central lesion. The patient was started on carbamazepine with some improvement of her symptoms after 6 months of follow-up.

## Discussion

### Definition

Auditory hallucinations are subjective auditory experiences without external auditory stimuli, and are categorized as elementary or complex hallucinations. Tinnitus and buzzing are considered elementary hallucinations, while the perception of words, voices, and music are classified as complex hallucinations (5).

### Etiology

Musical hallucinations still represent a disorder without clear etiology. Several in-depth reviews in the last three decades have been done in an attempt to synthesize multiple case reports, as well as to provide insight from basic neurosciences. Evers et al. reviewed 132 patients suffering from musical hallucinations (6), which included cases previously reported by Keshavan and colleagues (7).

Musical hallucinations usually occur in individuals of advanced age, although there are reports of patients in their 20s. Several conditions are considered potential etiologic or predisposing factors, including hearing impairment, psychiatric disorders, focal brain lesions, generalized brain atrophy, epilepsy, and intoxications.

Hearing impairment is the most commonly identified predisposing condition. However, moderate to severe hearing impairment is not a sufficient pathophysiological mechanism, since only a minority of these patients develops musical hallucinations. Psychiatric illnesses, including depression, schizophrenia, and obsessive-compulsive disorder have been associated with musical hallucinations (3). Social isolation is an additional contributing factor (8). Musical hallucinations have been reported in patients with brainstem hemorrhages, metastases, abscesses, arteriovenous malformations, and *Listeria* rhombencephalitis (9–11). Likewise, musical hallucinations have been associated with focal cortical lesions, global atrophy, and Hashimoto encephalopathy (12, 13). Musical hallucinations have been commonly reported among patients with epilepsy (6). An illustrative example was a patient with long-standing episodic abdominal pain followed by musical hallucinations associated with left temporo-parieto-frontal epileptiform discharges (14). Moreover, multiple medications, including opioids, tramadol, benzodiazepines, tricyclic antidepressants, carbamazepine, phenytoin, cannabinoids, ketamine, beta-blockers, among others, were found to be associated with musical hallucinations, as outlined by Prommer (15), who raised the possibility of an opioid-related modulation of auditory pathways as a possible pathophysiological mechanism of opioid-induced musical hallucinations.

### Clinical presentation

Musical hallucinations usually present with well-known tunes. Warner et al. reported a series of 30 consecutive patients with musical hallucinations over a 15-year period (16). Twenty-six patients experienced familiar tunes. Two-third of cases were religious hymns with “Abide with me” being the most common. Interestingly, there are reports of musical hallucinations representing novel musical motifs. Warren et al. reported the case of an 83-year-old musician who was able to notate her hallucinations – these motifs were not part of any known musical piece (17). The famous composers, Robert Schumann (1810–1856) and Bedrich Smetana (1824–1884), had attacks of vertigo and tinnitus with subsequent musical hallucinations (18). Schumann incorporated musical hallucinations on his Violin Concerto in D minor (19).

### Pathophysiology

Anatomic studies of music perception in humans demonstrate distinct brain regions involved in processing of different aspects of music – pitch, timber, temporal structure, and emotion (19). Computational models have been proposed to explain the modularity of music processing (20). Cortical auditory processing starts in the primary auditory cortex (PAC) located in the medial part of Heschl’s gyrus (HG). The secondary auditory cortical (SAC) area is located in the antero-lateral aspect of HG. Posterior to HG, is the planum temporale (PT), another associative area involved in auditory processing. Pitch analysis, timber perception, and temporal structure of music activate the anterior and posterior superior temporal lobe, right frontal operculum, lateral and mesial frontal cortices, basal ganglia, and cerebellum. Blood et al. studied the emotional perception of music employing PET, showing that pleasurable music activates the ventral striatum, midbrain, amygdala, orbito-frontal cortex, and ventral medial prefrontal cortex (21). These brain regions are known to be involved in reward and pleasure systems.

Griffiths employed PET imaging during active musical hallucinations and showed that the posterior aspect of the temporal lobes, right basal ganglia, inferior frontal cortices, and cerebellum were active during musical hallucinations, while PAC was not (22).

Since most patients with musical hallucinations experience familiar and a well-known music, it may be assumed that the underlying processes represent abnormal activation of a musical memory circuitry. Musical memory can be divided into short and long-term, where long-term memory can be further subdivided into explicit and implicit (23). Explicit long-term memory consists of semantic (ability to recognize music) and episodic memory (ability to remember the spatio-temporal personal and emotional context of music). Conversely, implicit memory includes priming and procedural memory (remembering skills to play music or a musical instrument). Several studies have examined the neural correlates of music memory by analyzing musical memory deficits in patients with specific brain lesions, as well as testing musical imagery and recognition by functional brain imaging. Both temporal lobes are necessary for learning and recognition of unfamiliar tunes (24). Patients who underwent PET scanning while imagining familiar tunes were found to have right temporal lobe and both frontal lobes activation during the process (25). Studies employing functional magnetic resonance imaging (fMRI) during music recognition tasks have shown similar results (26, 27). A case report by Cuddy et al. in a patient with Alzheimer’s dementia who had preserved responses to familiar music while other cognitive abilities were severely impaired, further supports the distinctiveness of musical memory neural substrate (28).

These data further support the emerging concept that musical hallucinations are result of a “deafferentation and release” phenomenon causing abnormal activity in otherwise normal segments of cortical music processing modules. Deafferentation with subsequent release is proposed to occur due to decreased cortical auditory input: hearing impairment, structural or chemically modulated disconnection within the auditory pathways, and abnormal excitation of cortical musical processing modules, among others.

### Treatment

There is no curative treatment for musical hallucinations at present time. Current treatment approaches are based on either removing potential inciting factors (hearing aids, treating underlying psychiatric disorder, stopping suspected causative medication, treating epileptic seizures, removing focal lesions if possible) or starting one of the medications reported to improve symptoms (6). Medications noted to ameliorate musical hallucinations include certain antipsychotics (olanzapine and quetiapine), antidepressants (fluvoxamine and clomipramine), antiepileptic medications (carbamazepine and valproate), and donepezil (29).

### Conclusion

A unique feature of our patient was the fact she was able to hum the tunes and retrieve lyrics to certain extent of non-recognizable songs. This represents partial semantic and implicit procedural musical memory retrieval since songs were recognized by her family members as popular songs. It also demonstrated that our patient did not experience novel music during her hallucinations. Since musical hallucinations caused partial retrieval of semantic, as well as procedural musical memory content of songs that our patient was not able to recognize (“forgotten” music), we propose the possibility of musical memories being present, but not accessible. It is also possible that our patient had fragmented preservation of musical memories, with key portions of those memories lost, precluding recognition. We find this proposition less likely since our patient would recognize music as familiar once it was played to her. Further research is necessary on the mechanisms of forgetfulness; in other words is forgotten information lost, or just not accessible? Although the former viewpoint is more prevalent at present time (30), the latter view would support attempts to retrieve “forgotten” memories in selected cases.

## Conflict of Interest Statement

The authors declare that the research was conducted in the absence of any commercial or financial relationships that could be construed as a potential conflict of interest.
